# Acetylcholinesterase inhibitor exposures as an initiating factor in the development of Gulf War Illness, a chronic neuroimmune disorder in deployed veterans

**DOI:** 10.1016/j.neuropharm.2020.108073

**Published:** 2020-04-02

**Authors:** Lindsay T. Michalovicz, Kimberly A. Kelly, Kimberly Sullivan, James P. O’Callaghan

**Affiliations:** a Health Effects Laboratory Division, Centers for Disease Control and Prevention – National Institute for Occupational Safety and Health, Morgantown, WV, USA; b School of Public Health, Boston University, Boston, MA, USA

**Keywords:** Gulf war illness, Acetylcholinesterase inhibitor, Neuroimmune, Neuroinflammation

## Abstract

Gulf War Illness (GWI) is a chronic multi-symptom disorder, characterized by symptoms such as fatigue, pain, cognitive and memory impairment, respiratory, skin and gastrointestinal problems, that is experienced by approximately one-third of 1991 Gulf War veterans. Over the nearly three decades since the end of the war, investigators have worked to elucidate the initiating factors and underlying causes of GWI. A significant portion of this research has indicated a strong correlation between GWI and exposure to a number of different acetycholinesterase inhibitors (AChEIs) in theater, such as sarin and cyclosarin nerve agents, chlorpyrifos and dichlorvos pesticides, and the anti-nerve agent prophylactic pyridostigmine bromide. Through studying these exposures and their relationship to the symptoms presented by ill veterans, it has become increasingly apparent that GWI is the likely result of an underlying neuroimmune disorder. While evidence indicates that AChEIs are a key exposure in the development of GWI, particularly organophosphate AChEIs, the mechanism(s) by which these chemicals instigate illness appears to be related to “off-target”, non-cholinergic effects. In this review, we will discuss the role of AChEI exposure in the development and persistence of GWI; in particular, how these chemicals, combined with other exposures, have led to a chronic neuroimmune disorder.

This article is part of the special issue entitled ‘Acetylcholinesterase Inhibitors: From Bench to Bedside to Battlefield’.

## 1. Introduction

Acetylcholinesterase inhibitors (AChEIs) comprise a class of chemicals that can be generally categorized as therapeutics, pesticides, or nerve agents. During the 1991 Gulf War, soldiers had the potential to be exposed to all three of these categories of AChEIs: the pesticides chlorpyrifos (CPF) and dichlorvos (DDVP) were regularly and pervasively used for pest control, pyridostigmine bromide (PB) was prescribed to be taken every 8 h as a prophylactic against potential nerve agent exposure, and the demolition of ammunition storage facilities released sarin and cyclosarin nerve agents and potentially exposed veterans to these chemical weapons (Tuovinen et al., 1999; Institute of Medicine (US) Committee on Health Effects Associated with Exposures During the Gulf War, 2000; Research Advisory Committee on Gulf War Veteran’ Illnesses, 2008; White et al., 2016). Exposure to AChEIs has been repeatedly implicated as the potential cause of Gulf War Illness (GWI), a chronic multi-symptom disorder affecting nearly one-third of the veterans that returned from the 1991 conflict, by both epidemiological (Sullivan et al., 2003; Research Advisory Committee on Gulf War Veteran’ Illnesses, 2008; Steele et al., 2012; Kerr, 2015; White et al., 2016; Sullivan et al., 2018) and preclinical studies (Henderson et al., 2001; Amourette et al., 2009; Lamproglou et al., 2009; Abdullah et al., 2012; Abdullah et al., 2013; Parihar et al., 2013; Hattiangady et al., 2014; Ojo et al., 2014; Nutter et al., 2015; O’Callaghan et al., 2015; Zakirova et al., 2015; Abdullah et al., 2016; Cooper et al., 2016; Phillips and Deshpande, 2016; Pierce et al., 2016; Alhasson et al., 2017; Emmerich et al., 2017; Flunker et al., 2017; Locker et al., 2017; Shetty et al., 2017; Zakirova et al., 2017; Ashbrook et al., 2018; Carreras et al., 2018; Cooper et al., 2018; Koo et al., 2018; Macht et al., 2018; Miller et al., 2018; Phillips and Deshpande, 2018; Seth et al., 2018; Macht et al., 2019; Michalovicz et al., 2019). Acute exposure to toxic levels of AChEIs, particularly the organophosphate (OP) compounds like the pesticides and nerve agents, has a number of systemic effects (i.e. salivation, lacrimation, urination, defecation, gastrointestinal upset, emesis, miosis [SLUDGEM]) and can cause seizures. However, it is the long-term neurological symptoms associated with exposure to these compounds that harmonize with many of the long-lasting symptoms of GWI: fatigue, pain, mood disorders, cognitive and memory impairment (Fukuda et al., 1998; Steele, 2000; Haley et al., 2001; Golomb, 2008; Sullivan et al., 2003; Maule et al., 2018). Furthermore, the similarity of these symptoms to those of sickness behavior, the adaptive behavioral response elicited by illnessor infection-induced neuroinflammation (Dantzer and Kelley, 2007; Dantzer et al., 2008), has suggested that GWI is the result of an underlying chronic neuroimmune disorder; a hypothesis that has been supported by both preclinical animal (O’Callaghan et al., 2015; Zakirova et al., 2016; Alhasson et al., 2017; Locker et al., 2017; Shetty et al., 2017; Ashbrook et al., 2018; Carreras et al., 2018; Kodali et al., 2018; Koo et al., 2018; Macht et al., 2018, 2019; Miller et al., 2018; Hernandez et al., 2019; Joshi et al., 2019; Madhu et al., 2019; Michalovicz et al., 2019) and clinical studies (Broderick et al., 2013; Parkitny et al., 2015; White et al., 2016; Coughlin, 2017; Abou-Donia et al., 2017; Georgopoulous et al., 2017; Alshelh et al., 2020). In spite of the fact that the ACh signaling facilitated by AChE inhibition is typically anti-inflammatory (Pavlov et al., 2003; Pavlov and Tracey, 2005), several preclinical studies have directly shown a connection between GWI-relevant AChEI exposures and neuroinflammation (Ojo et al., 2014; O’Callaghan et al., 2015; Locker et al., 2017; Ashbrook et al., 2018; Koo et al., 2018; Miller et al., 2018). Moreover, while there are many hurdles to directly evaluate neuroinflammation in living humans, a recent clinical Positron Emission Tomography (PET) study found strong signals for the neuroinflammatory marker, 18 kDa transducer protein (TSPO), throughout the cortex of veterans with GWI compared to controls, providing the first direct, *in vivo* data to show neuroinflammation in the illness (Alshelh et al., 2020).

In this review, we will discuss the three major groups of AChEIs that soldiers were exposed to during the 1991 Gulf War that have been associated with GWI: nerve agents, pesticides, and pyridostigmine bromide. In particular, we will focus on discussing the preclinical body of data that has supported the hypothesis that GWI is the result of an underlying neuroinflammatory/neuroimmune disorder. Lastly, we will discuss emerging evidence that suggests that this neuroimmune dysfunction is the result of persistent, non-cholinergic effects of AChEI exposure.

### 1.1. Acetylcholinesterase inhibitor exposures in GWI

The emergence of a chronic multi-symptom disorder in veterans immediately following the 1991 Gulf War raised questions regarding the possibility that harmful exposures occurred during the war (Pennisi, 1996). Soldiers that were deployed during the 1991 Gulf War were exposed to a number of conditions that could have increased their risk for negative health impacts, including pesticides, nerve agent, oil well fire smoke, depleted uranium munitions, prophylactic drugs and vaccinations, paints, and psychological and/or physiological stress (White et al., 2016). However, the prevalence of neurological and neuropsychological symptoms among veterans with GWI has fueled an interest in the neurotoxicant exposures that may have been experienced in theater (White et al., 2016). Among these neurotoxicants, there was the potential for and, in some cases, documentation of, the exposure of soldiers to both irreversible and reversible AChEIs during deployment. The delineation between these two subcategories of AChEIs is important because the perceived risks associated with irreversible OP AChEIs, i.e. those pesticides and nerve agents that permanently bind and inactivate the enzyme, is greater than the reversible AChEIs (including pharmaceuticals) that only modulate enzyme activity through on-off binding dynamics. In this section, we will discuss both subclasses of AChEIs that have been investigated in GWI and how exposure to these compounds may contribute to the associated chronic neuroinflammatory dysfunction.

### 1.2. Sarin, cyclosarin, and nerve agent surrogates

Exposure to sarin and/or cyclosarin nerve agent, both OP AChEIs, was a very real risk to soldiers deployed during the 1991 Gulf War. Interviews with veterans of the 1991 Gulf War have indicated that many of them recall hearing chemical alarms in camp during deployment (Haley and Kurt, 1997; Kurt, 1998; Haley and Tuite, 2013; Chao et al., 2016). Additionally, several investigations have determined that the destruction of multiple munitions storage sites by U.S. troops released a plume containing both sarin and cyclosarin that is predicted to have exposed upwards of 100,000 soldiers (Institute of Medicine (US) Committee on Health Effects Associated with Exposures During the Gulf War, 2000; Research Advisory Committee on Gulf War Veteran’ Illnesses, 2008). Interestingly, a survey of soldiers that were in proximity to one of these detonation sites, Khamisiyah, found that the majority of respondents did not report any acute cholinergic symptoms (Institute of Medicine (US) Committee on Health Effects Associated with Exposures During the Gulf War, 2000). However, many studies of sick veterans have uncovered an association between potential nerve agent exposures and GWI presentation (see review in Golomb, 2008; White et al., 2016). While nerve agent exposure has been associated with incidence of high blood pressure, diabetes, arthritis and chronic bronchitis in GWI (Zundel et al., 2019), the observations of MRI-assessed brain structural abnormalities like decreased gray and white matter volumes and increased ventricle size (Heaton et al., 2007; Chao et al., 2010, 2011, 2014, 2015; Chao and Zhang, 2018), as well as poor neurobehavioral performance (White et al., 2001; Proctor et al., 2006; Toomey et al., 2009) and an increased risk for brain cancer (Bullman et al., 2005) are more indicative of the long-term brain effects of nerve agent exposure. These studies combined with the result of the Department of Defense survey suggest that GWI may be the result of low-level exposures to sarin and/or cyclosarin that went undetected due to a lack of immediate/acute neurotoxic effects.

Though posing an obvious neurotoxicological risk, only a handful of preclinical studies have investigated the role of potential sarin exposure in the development of GWI. A few of these studies have investigated the potential outcomes of low-dose, sub-lethal exposure(s) to sarin or sarin surrogates alone in relationship to GWI symptomology. While subclinical inhalation of sarin has been demonstrated to be capable of producing signs of immunosuppression, such as reduced T-cell responses and reduced corticosterone levels (Henderson et al., 2001; Kalra et al., 2002), as well as autonomic imbalance and cardiomyopathy (Shewale et al., 2012), these studies do not directly correlate with the neuroimmune dysfunction hypothesis for GWI. However, repeated, low-dose exposure to the sarin surrogate diisopropyl fluorophosphate (DFP) has been shown to instigate depression, cognitive impairment, and neuronal damage (Phillips and Deshpande, 2016, 2018). In these studies, rats were injected with 0.4–0.5 mg/kg, s.c. DFP once a day for 5 days and, when assessed three to six months after exposure, demonstrated memory impairments in novel object recognition and object location tasks, signs of anxiety and depression as indicated by increased immobility time in the forced swim test, reduced sucrose preference, and reduced time in the open arms of the elevated plus maze. These behavioral observations were concurrent with increased levels of neuronal calcium and neuronal cell damage in the hippocampus. In addition, 14-day exposure to the same dose of DFP was found to result in impaired axonal transport (Naughton et al., 2018). While there were no direct evaluations of neuroinflammation or neuroimmune dysfunction in these studies, an expanding body of research has suggested an association between neuroinflammation and depression (Walker et al., 2013), as well as axonal transport deficits (Errea et al., 2015). However, these results not only support a role for nerve agent exposure in the development of GWI symptoms, but also demonstrate that these low-dose exposures are capable of producing long-lasting effects that can translate to the decades of persistent illness experienced by veterans with GWI.

Deployed soldiers were exposed to significant physiological stressors such as exercise and extreme temperatures (Young et al., 1992; Sapolsky, 1998; Sullivan et al., 2003) that had the potential to interact with or modulate the responses to chemical exposures. As such, several studies investigating the contribution of nerve agent exposure to GWI have found positive correlations to illness symptomology when sarin surrogates have been combined with a stressor (O’Callaghan et al., 2015; Locker et al., 2017; Ashbrook et al., 2018; Koo et al., 2018; Miller et al., 2018; Craddock et al., 2018; Michalovicz et al., 2019; Belgrad et al., 2019). In this GWI rodent model, a single dose of DFP is preceded by a chronic (4–7 day) exposure to exogenous corticosterone (CORT) provided in the drinking water (200 mg/L) to mimic the high physiological stress experienced by soldiers during deployment. Though currently acute in scope, the extensive evaluation of this model has strongly supported the role of neuroimmune dysfunction as the underlying cause of GWI showing that this combination of exposures results in CORT priming of the DFP neuroinflammatory response leading to significantly increased inflammatory cytokine mRNA throughout the brain, including cortex, hippocampus, and striatum (O’Callaghan et al., 2015; Locker et al., 2017; Koo et al., 2018; Miller et al., 2018), along with alterations in neuroimmune signaling via histone modification and DNA methylation changes (Ashbrook et al., 2018) shortly after the exposures. Furthermore, evaluation of this paradigm using a literature-derived, logic model of neuron-glia interactions indicated the potential for GWI to derive from an aberrant homeostatic neuroinflammatory profile (Craddock et al., 2018). Notably, these neuroinflammatory effects occur in the absence of significant peripheral inflammation (Michalovicz et al., 2019). While DFP alone has some minor proinflammatory effects in the liver and serum, these effects are largely suppressed by the prior CORT exposure, findings that differentiate them from the CORT priming of the DFP response in the brain and highlight the role of neuroinflammation in GWI. Interestingly, similar results were found in a clinical evaluation of neuroinflammation where veterans with GWI presented with an increased neuroinflammatory PET signal in the cortex in spite of there being no difference in plasma cytokine levels in comparison to healthy controls (Alshelh et al., 2020).

While no changes have been observed in astrocytes or microglia, the brain’s primary immune cells, in the acute time points following exposure in this model (O’Callaghan et al., 2015), Belgrad et al. (2019) found that DFP alone had effects on oligodendrocytes, another glial cell type with immune function (Peferoen et al., 2014). In this study, DFP exposure decreased the number of mature and proliferating oligodendrocytes in the rat cortex and corpus callosum out to 21 days post-exposure while combined CORT and DFP exposure ameliorated these effects. However, this combined exposure resulted in an increase in myelin basic protein, a protein crucial to proper myelin structure, that may be indicative of demyelination or injury (Kristensson et al., 1986; Bartholdi and Schwab, 1998). As such, Naughton et al. (2018) found that their chronic DFP exposure paradigm resulted in disordered, de-compacted myelin sheaths. These exposures have also been translated into neuronal cell culture and shown to affect microtubule acetylation and axonal transport of mitochondria (Rao et al., 2017). In addition to these cellular and molecular level changes, high-order diffusion MRI of GWI rat brains demonstrated alterations in brain structure and connectivity concurrent with neuroinflammation that may be indicative of subtle structural changes in dendrites or glial processes (Koo et al., 2018). Taken at early time points following GWI exposure, these diffusion changes may capture the initiating conditions that have led to the more significant changes in brain structure reported by traditional MRI in veterans with GWI (Heaton et al., 2007; Chao et al., 2010, 2011, 2014, 2015; Chao and Zhang, 2018), and suggests neuroinflammation as an underlying cause.

While both sarin and DFP can significantly increase brain ACh levels by inhibiting AChE activity, the exacerbated neuroinflammation instigated by corticosterone priming of DFP exposure was associated with a reduction in ACh levels and mitigation of AChE inhibition in the brain (Locker et al., 2017; Miller et al., 2018). These studies provide strong evidence for a causative association between nerve agent and wartime stress exposure and GWI, suggesting that these exposures have resulted in a persistent shift in how the neuroimmune system functions; ultimately, allowing for a chronic neuroinflammatory state that underlies the neurological and systemic issues experienced by the ill veterans. These results also propose a mechanism by which a condition of high physiological stress facilitated the circumvention of the anti-inflammatory cholinergic signaling pathway. Furthermore, the other studies discussed here (Phillips and Deshpande, 2016, 2018; Naughton et al., 2018) used low doses of DFP that did not produce acute cholinergic crisis. Though the potential for veterans with GWI to have been exposed to chemical weapons containing nerve agent has been highly controversial in the past, studies that have investigated these exposures at low doses, alone or in combination with the high physiological stress experienced in theater, have found compelling evidence for the involvement of nerve agent in the development of a chronic neuroimmune disorder underlying GWI. Moreover, the recent evidence indicating that wartime stress may have reduced the anticholinergic effects of these agents (Locker et al., 2017; Miller et al., 2018) suggests that other, non-cholinergic mechanisms are likely responsible for GWI (Terry, 2012; Naughton and Terry, 2018).

### 1.3. Chlorpyrifos (CPF) and dichlorvos (DDVP)

In addition to potential low-dose nerve agent exposure, frequent and pervasive pesticide usage also constituted a repeated, daily exposure in theater that was employed to help prevent vector-borne illnesses. According to the Environmental Exposure Report on Pesticides, soldiers were exposed to pesticides via treated uniforms and tents, flea collars, pest strips, fogging, and personal application with an estimated 41,000 having been overexposed (Winkenwerder, 2003); these exposures have been associated with GWI (Haley and Kurt, 1997; Golier et al., 2007; Golomb, 2008; Steele et al., 2012; White et al., 2016; Sullivan et al., 2018). Among these pesticides, veterans with GWI were exposed to the OP AChEIs, CPF and DDVP. Highlighting these exposures, a recent study of GW military pesticide applicators indicated a strong association between cognitive impairments and higher levels of exposure to DDVP pest strips (Sullivan et al., 2018). Unfortunately, to our knowledge, there has been no direct evaluation of GWI-related DDVP exposure in animal models, but DDVP exposure has the potential to produce neuroimmune responses such as microglial activation, increased inflammatory cytokine expression and neurodegeneration (Kaur et al., 2007; Binukumar et al., 2011). However, several studies have investigated the potential role of CPF exposure in GWI and found significant neurological effects (Ojo et al., 2014; Hernandez et al., 2015; Nutter et al., 2015; Cooper et al., 2016, 2018; Flunker et al., 2017; Locker et al., 2017; Miller et al., 2018). Among these studies, investigators have demonstrated that repeated exposure to CPF can cause persistent impairment of axonal transport (Hernandez et al., 2015), loss of synaptic integrity and neurogenesis in the hippocampus (Ojo et al., 2014), and decreased pain threshold when combined with PB, PER and DEET (Nutter et al., 2015; Cooper et al., 2018; Flunker et al., 2017; Cooper et al., 2018, 2018). While these results do not directly support the neuroimmune dysfunction hypothesis of GWI, conditions like neuropathic pain, impaired hippocampal neurogenesis, and axonal transport deficits have been associated with neuroinflammation (Ellis and Bennett, 2013; Walker et al., 2013; Errea et al., 2015; Valero et al., 2017). However, a few studies have directly evaluated neuroimmune-related consequences of CPF exposure. Specifically, it has been shown that exposure to the active metabolite of CPF, chlorpyrifos oxon (CPO; 8 mg/kg, i.p.), results in exacerbated neuroinflammation in mice when combined with prior exogenous exposure to the stress hormone CORT as indicated by an increase of brain inflammatory cytokine mRNA across different brain areas (Locker et al., 2017; Miller et al., 2018). Chronic exposure to CPF alone or in combination with PB and the pesticide permethrin (PER) in mice for 10 days was found to cause an increase in GFAP, indicative of neuroinflammation-associated astrocyte activation; these results were brain region specific, with CPF alone increasing GFAP in motor cortex and hippocampus and CPF + PB + PER combined exposure causing astrogliosis in the piriform cortex and basolateral amygdala (Ojo et al., 2014). Furthermore, similar to the results seen with the sarin surrogate DFP, prior stressor exposure mitigated brain AChE inhibition and decreased ACh levels instigated by CPF exposure (Locker et al., 2017; Miller et al., 2018). While these studies highlight a role for OP AChEI pesticides in the neuroimmune dysfunction associated with GWI, more investigation is needed to understand the mechanisms by which they instigate illness (i.e. non-cholinergic pathways).

### 1.4. Pyridostigmine bromide (PB)

The requirement for soldiers deployed during the 1991 Gulf War to take prophylactic doses of PB in hopes of preventing serious complications from potential nerve agent exposures has made the drug a prime target for investigation in GWI. As a reversible AChEI with minimal permeability across the blood brain barrier (BBB), little risk was expected from prophylactic treatment with PB particularly considering the dire consequences of nerve agent exposure. Substantiating this notion is the prevalence of reversible AChEIs as pharmacological agents for the treatment of several illnesses, including myasthenia gravis, Alzheimer’s disease, glaucoma, and others (Čolović et al., 2013). However, as early as 1997, epidemiological studies began to uncover an association between chemical exposures, including PB, and the emerging illness in a large population of veterans (Haley et al., 1997a; Haley et al., 1997b; Haley and Kurt, 1997; The Iowa Persian Gulf Study Group, 1997). Over the years, these initial studies have been expanded to reveal strong correlations between PB exposure alone or in combination with other chemicals and the various symptoms of GWI (Kurt, 1998; Nisenbaum et al., 2000; Schumm et al., 2001; Wolfe et al., 2002; Sullivan et al., 2003; Golomb, 2008; Research Advisory Committee on Gulf War Illnesses, 2008; Steele et al., 2012; Steele et al., 2015; White et al., 2016; Sullivan et al., 2018; Zundel et al., 2019). In particular, a few studies found a positive correlation between the number of PB pills taken and the severity of individual GWI cases (Wolfe et al., 2002; Lucas et al., 2007; Golomb, 2008). While most studies focused on evaluating GWI as the collection of its symptoms, a few studies have found specific associations between GWI, PB use, and neuromuscular dysfunction and suppressed cortisol responses (Golier et al., 2006, 2007), as well as gene-exposure interactions with butyrylcholinesterase genotypes (Steele et al., 2015) and increased risk of heart attack and diabetes (Zundel et al., 2019).

While these clinical findings have driven numerous preclinical studies to investigate the role of PB exposures in GWI, there has been minimal evidence to support its involvement in the underlying neuroimmune dysfunction associated with the illness. As such, when PB has been evaluated alone without exposure to any other mediating factors, the drug has minimal deleterious effects on mice or rats (Abou-Donia et al., 1996; Amourette et al., 2009; Lamproglou et al., 2009; Barbier et al., 2009; Locker et al., 2017; Macht et al., 2018; Hernandez et al., 2019). While very few studies have looked directly at neuroinflammation as a result of PB exposure, those that have investigated this directly, found very minimal proinflammatory effects of the drug with an inclination towards anti-inflammatory outcomes (Locker et al., 2017; Hernandez et al., 2019). Moreover, while it has been suggested that stress may increase the permeability of the BBB allowing for PB to gain access to the brain (Friedman et al., 1996; Hanin, 1996; Shen, 1998; Shaikh et al., 2003), several studies investigating the possibility that wartime stress affected the brain accessibility of PB have found that multiple stressor methods do not increase BBB permeability, affect PB’s reduction of brain ChE activity, nor elicit the elaboration of inflammatory markers in the brain or blood in animal models of GWI (Sinton et al., 2000; Song et al., 2002; Tian et al., 2002; Amourette et al., 2009; Locker et al., 2017; Macht et al., 2018). These results suggest that the relationship between PB exposure and GWI that has been supported by epidemiological studies is not straightforward but does not seem to support a role for PB alone in the neuroimmune dysfunction hypothesis.

#### 1.4.1. PB in combination with other GW-relevant chemical exposures

As stated previously, veterans suffering with GWI had the potential to be exposed to many chemicals in theater. As such, a recent study of GW military pesticide applicators found a strong association between combined high pesticide and PB exposure and greater cognitive impairment along with higher rates of GWI (Sullivan et al., 2018). Other studies involving co-exposures to PB and sarin had mixed results. Abou-Donia et al. (2002) found that while PB offered some peripheral ChE protection it did not mitigate sarin-reduced brain AChE activity. Furthermore, while all exposure combinations (PB alone, sarin alone, PB + sarin) caused worsened sensorimotor impairments compared to controls, as assessed by grip strength and beam- and inclined plane-walking (Abou-Donia et al., 2002), the results were largely dependent on the dosage of sarin given and the amount of time following exposure. Specifically, combined exposure with PB was detrimental with higher dosages of sarin for the beam-walk score and degree at which slipping occurred on the inclined plane, but protective for the same tasks when combined with lower dosages of sarin. A separate study found that PB protected against short-term sarin-induced neurobehavioral impairment, as indicated by enhanced acoustic startle response and anxiety/decreased habituation in the open field test, while PB + sarin increased pain threshold at 16 weeks after exposure (Scremin et al., 2003). However, it is difficult to compare the results between these two studies as they used drastically different exposure models.

In addition, a significant number of exposure models have been developed that combine PB with other, non-AChEI, GW-relevant chemicals including: permethrin (PER) with or without stress (Abdullah et al., 2011; Zakirova et al., 2015; Alhasson et al., 2017; Nizamutdinov et al., 2018; Seth et al., 2018; Joshi et al., 2019); PER and DEET with or without stress (Abou-Donia et al., 1996; Abdel-Rahman et al., 2002; Abdullah et al., 2012; Parihar et al., 2013; Hattiangady et al., 2014; Megahed et al., 2015; Pierce et al., 2016; Shetty et al., 2017; Carerras et al., 2018; Petrescu et al., 2018; Madhu et al., 2019); PER and CPF with or without DEET (Ojo et al., 2014; Nutter et al., 2015; Cooper et al., 2016, 2018; Flunker et al., 2017). The prominence of these mixtures in GWI models, which largely revolve around combined exposures with PER and DEET, stem from a recommendation in the report from the Institute of Medicine (US) Committee to Review the Health Consequences of Service During the Persian Gulf War (1995) and an initial study of these chemicals in a hen model (Abou-Donia et al., 1996). While some of these models have demonstrated neuroimmune-related effects, these models generally evaluate conditions only when all chemical exposures are combined and, therefore, the results are likely due to the other chemicals employed in these models rather than PB. However, one study presented by Cooper et al. (2018) found that inclusion of PB in their combination exposure with permethrin, chlorpyrifos, and DEET was requisite for the development of chronic pain and aberrant nociceptor signaling. Overall, the disparity in positive results between models that more directly investigate the role of PB in GWI and those that use combinations with other chemicals like sarin, PER, and/or CPF further highlights the likelihood that PB had a modulatory effect on the response to these other exposures in the initiation of GWI.

## 2. How did acetylcholinesterase inhibitor exposures cause the neuroimmune dysfunction associated with GWI?

While it has been suggested that there is a strong connection between AChEI exposures and the development of GWI and these chemicals have been demonstrated to have numerous cellular, biochemical, physiological, and neuropsychological effects, the direct mechanism by which exposure has resulted in this chronic illness has remained obscure. As summarized in this review, we suggest that the literature indicates that OPs that irreversibly inhibit AChE, like sarin and its surrogates, chlorpyrifos, and dichlorvos, seem to be the key exposure for the development of neuroimmune dysfunction in GWI. Under normal conditions, OP AChEIs exert their toxicological effects by organophosphorylating and inactivating the cholinesterase enzymes which produces acute toxicity. However, it is unclear whether exposed veterans with GWI exhibited any signs of acute AChEI intoxication. As far as potential sarin or cyclosarin exposure is concerned, the survey conducted of soldiers within the vicinity of the Kamisiyah detonation suggests that they did not experience signs of acute toxicity (Institute of Medicine (US) Committee on Health Effects Associated with Exposures During the Gulf War, 2000), suggestive of low-level exposure. As discussed in this review, a number of studies have recapitulated GWI in animal models using low doses of sarin, sarin surrogates, or pesticides. Similarly, it has been shown that under conditions of high physiological stress, the cholinergic toxicity of agents like DFP and CPO is suppressed as evidenced by a reduction in the percentage of enzyme inhibition and amelioration of the increase in brain ACh (Locker et al., 2017; Miller et al., 2018). We hypothesize that these conditions, e.g. low-level exposures alone or combined with high physiological stress, avoid the acute cholinergic toxicity of the OP AChEIs due to a dose-dependent interaction with the enzyme itself. In the case of low level exposure, the doses of AChEIs encountered may be below threshold for significant AChE inhibition. When considering the interaction of AChEI exposures and high physiological stress, studies have shown that stress or CORT exposures can increase AChE activity (Oriaku and Soliman, 1986; Fatranska et al., 1987; Tsakiris and Kontopoulos, 1992); thus, altering the activity of AChE in the tissue has the potential to compensate for the inhibition instigated by AChEI exposure by normalizing ACh levels. Therefore, in both of these conditions, exposure to OP AChEIs would have minimal functional impact on cholinergic signaling and not trigger the prototypical cholinergic anti-inflammatory signaling pathway (Pavlov et al., 2003; Pavlov and Tracey, 2005); facilitating the significant neurological results demonstrated following OP AChEI exposure, including signs of neuroimmune dysfunction, such as increased expression of neuroinflammatory cytokines and astrogliosis (Ojo et al., 2014; Locker et al., 2017; Miller et al., 2018). Thus, we suggest that GWI may be the result of aberrant neuroimmune signaling that is instigated by the organophosphorylation of non-cholinesterase targets by the OP AChEIs, a hypothesis that has been previously suggested (Grigoryan et al., 2008, 2009; Terry et al., 2012; Naughton and Terry, 2018) and warrants further investigation in relationship to neuroinflammatory signaling.

## 3. Conclusions

**Fig. 1.**
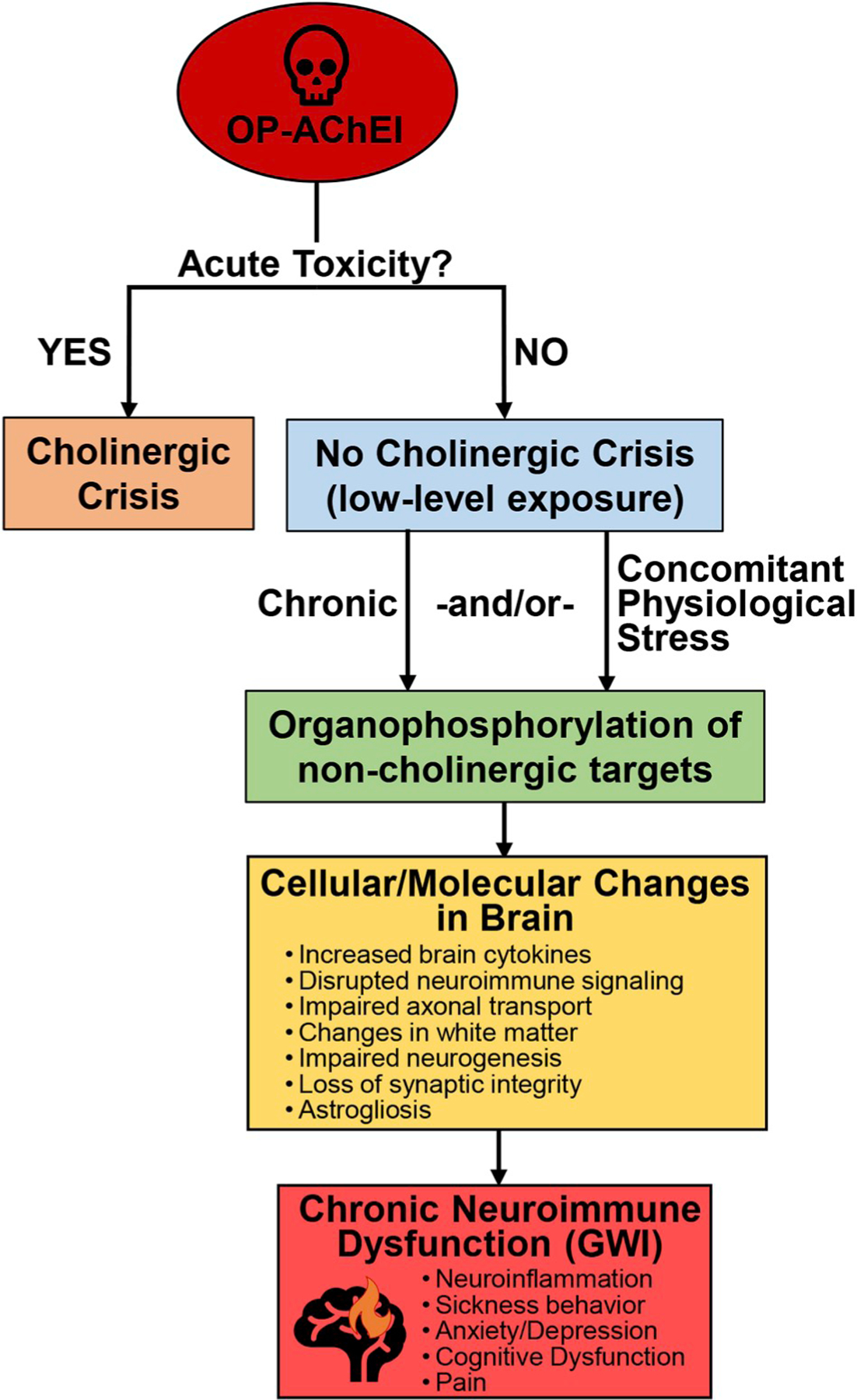
Mechanism of acetylcholinesterase inhibitor-induced neuroimmune dysfunction in Gulf War Illness. Acetylcholinesterase inhibitors (AChEIs), in particular the organophosphate (OP) chemicals, can instigate illness in two ways: (1) acute toxicity that results in cholinergic crisis (salivation, lacrimation, urination, defecation, gastrointestinal upset, emesis, miosis [SLUDGEM]; seizures) and carries a higher risk of mortality; (2) long-term illness in the absence of an acute cholinergic crisis. The latter condition is proposed to be the result of chronic low-level AChEI exposure with or without concurrent exposure to physiological stress. The myriad of cellular and molecular effects that have been demonstrated in the brain as a result of these exposures are hypothesized to be the consequences of organophosphorylation of non-cholinergic targets, e.g. neuroinflammatory signaling mediators. Ultimately, these effects culminate into a state of chronic neuroimmune dysfunction, the underlying cause of Gulf War Illness (GWI).

As stated throughout this review, GWI is believed to be a result of a combination of exposures/conditions that were experienced by soldiers during deployment. In the years that have followed the 1991 Gulf War, research has suggested that AChEIs are chief among these exposures as culprits in the development of GWI, particularly when combined with stressors to mimic the extreme conditions experienced by soldiers during deployment. Overall, the irreversible, OP AChEIs, both nerve agents and pesticides, are more likely to have played a primary role in the development of GWI as they show a strong correlation with the neuroimmune dysfunction suspected to be the underlying cause of illness among these veterans. While the administration of PB pills has long been suspect in the development of GWI, our review of the literature has suggested that the correlation between PB and GWI is not strongly supported by preclinical investigations using animal models of exposure; thus, the role of PB in GWI remains unclear. In spite of their common cholinergic functions, we strongly suspect that GWI is the result of the actions of OP AChEI exposures on non-cholinergic targets (Grigoryan et al., 2008, 2009; Terry et al., 2012; Locker et al., 2017; Miller et al., 2018; Naughton and Terry, 2018), namely organophosphorylation of proteins that are crucial to neuroinflammatory signaling. Long-term low dose inhibition of AChE by these compounds and other contributing factors, such as physiological stress and other chemical exposures, may have played a role as well to facilitate the development of a chronic neuroimmune disorder (Fig. 1). By the continued investigation into potential mechanisms underlying GWI pathobiology, we may be able to uncover therapeutic targets that can be modulated to more successfully treat GWI symptoms and/or reverse the underlying aberrant neuroimmune function associated with this disorder.

## HIGHLIGHTS

- Gulf War Illness (GWI) is associated with underlying neuroimmune dysfunction.
- Veterans with GWI were exposed to several acetylcholinesterase inhibitors (AChEIs).
- Organophosphate AChEIs, nerve agent and pesticides, can instigate neuroinflammation.
- While associated with GWI, pyridostigmine does not cause neuroinflammation.
- Lack of acute toxicity suggests GWI results from non-cholinergic actions of AChEIs.
